# Ant-Inspired Bionic Design Method for the Support Structure of the Fengyun-3 Satellite Payload Infilled with Lattice Structure

**DOI:** 10.3390/ma16020736

**Published:** 2023-01-11

**Authors:** Hang Li, Ruiyao Liu, Haijun Wang, Renlong Xin, Zhenbang Xu, Zhenglei Yu

**Affiliations:** 1 Key Laboratory of Engineering Bionics, Ministry of Education, Jilin University, Changchun 130022, China; 2 Changchun Institute of Optics, Fine Mechanics and Physics, Chinese Academy of Sciences, Changchun 130022, China; 3 Department of Mechanics, School of Mechanical and Aerospace Engineering, Jilin University, Changchun 130022, China; 4 China Key Laboratory for Cross-Scale Micro and Nano Manufacturing, Ministry of Education, Changchun University of Science and Technology, Changchun 130012, China

**Keywords:** lattice structure, structural bionic, lightweight design, topology optimization, aerospace support structure

## Abstract

Owing to their high design freedom and excellent performance, lattice structures have shown outstanding capabilities and great potential in aeronautics and astronautics fields. In this paper, we propose a method to construct lattice structures by parameterizing biological features. An ant-leg configuration is used as the bionic object to generate a bionic lightweight design with a gradient lattice structure. To achieve the above goal, an innovative optimization method combining topology optimization, size optimization, and a bionic lattice structure is proposed in this paper. Taking the support structure of the Fengyun-3 satellite payload as the research object, this optimization method is applied to optimize the design. Further, the reconstructed optimization model and the original model are simulated to evaluate and compare the structural performance. The simulation results show that when combined with bionic lattice structure and structural optimization, the method can achieve the lightweight design goal while ensuring the stiffness and strength of the structure. The results demonstrate that the application of a bionic lattice design in a lightweight design has feasibility and expectable potential.

## 1. Introduction

In the aerospace field, lightweight design is the orientation that scholars strive to pursue [1,2]. The support structure is the main load-bearing component of an aerospace load but also the component with the largest proportion of mass, generally accounting for 40–50% [3]. Therefore, the lightweight design of a support structure is particularly significant. Traditional lightweight design methods include topology optimization, shape optimization, and size optimization. Topology optimization is often used as a tool to find effective design concepts in the early design stage, while size optimization and shape optimization are tools for later detailed designs [4,5,6]. But the results of topology optimization are often complex and unmanufacturable. In consequence, the complexity of optimization results has become a barrier to processing and manufacturing [7,8,9].

As a lightweight structure, lattice provides high performance such as high specific stiffness, accompanied by a relatively low density and good energy absorption characteristics. A 3D lattice structure is believed to be a significant structure for weight reduction [10,11,12]. In the past few years, honeycomb cellular lattice has been regarded as the most classical lattice structure and applied in aerospace, medical, and engineering products [13,14,15]. The superior structural characteristics of truss lattice have also attracted the attention of many researchers. Wang et al. presented a composite sandwich structure with a pyramidal truss core, which had excellent mechanical behavior [16,17,18]. Rathbun et al. fabricated metallic sandwich panels with tetrahedral truss cores [19,20,21]. Nevertheless, the bending-dominated deformation of the lattice cannot fully utilize the load-carrying properties of materials [22,23,24]. Additionally, the lattice cores are liable to buckling under compressive loads, while the thick core leads to large spacing between nodes, which weakens the resistance to local panel buckling [25,26,27]. Although the development of additive manufacturing has broken the shackles of traditional manufacturing technology and provided opportunities for the production of lattice structures [28,29,30], owing to the structural defects and the challenges encountered in traditional manufacturing difficulties, the application of 3D lattice structures in aerospace and aeronautical support structures remains a significant challenge.

Over 500 million years of evolution, natural organisms have developed many structural types with excellent mechanical properties, thanks to selective pressures from their living environment [31,32,33]. For example, the internal gloss layer of many mollusk shells has excellent mechanical properties, and its hierarchical arrangement can be used as a guide for the design of high-strength and lightweight materials or structures [34]. In addition, deer antlers, bones, and teeth all show outstanding mechanical properties. Deer antler can endure bending without damage and has impact resistance [35]. Bones and teeth can maintain a large compressive force without bending or cracking [36,37,38]. Some efforts have been made to apply these advantages to the design of aircraft structures, including aircraft-reinforcing frames and small wings [39,40,41]. As a typical microhexapod structure, foraging workers of ants often carry food loads many times their body weight. The well-developed legs of ants show an excellent load-carrying ability, as well as the double improvement of strength and stability. Scholars have studied the biological characteristics of ants. The well-known size-grain hypothesis predicts that small ants should have smaller legs than larger ants, and ants that forage in flat environments are generally larger than those that forage in more-complex environments [42,43]. This configuration is very promising for supporting structures and can provide guidance for lightweight designs. However, as far as we know, there is no research on the application of ant carrying capacity in lightweight structure design.

In this article, 14 lattice structures are designed on the basis of the research of ant-leg configuration characteristics inspired by ants’ incredible carrying capacity. And their mechanical performance is verified by using the finite element method (FEM), conducting a vibration experiment, and conducting a compression experiment. Furthermore, the lightweight design of the support structure of the Fengyun-3 satellite payload is realized by integrating topology optimization, bio-inspired lattice structure filling, and size optimization. The configuration principles of ant legs are applied to fill part of the support structure. By comparing the stiffness and strength of the original model and the optimized model, it is proved that the proposed bionic lattice lightweight method has certain advantages, which will provide guidance for the lightweight design of support structures in the aerospace field.

## 2. Prototype Structure of Ants

### 2.1. Lectotype of Ants

Camponotus is the largest ant genus in the world, belonging to Formicinae. It prefers digging trees damaged by water and is good at carrying and hiking. Black-gold Turkish Camponotus is a typical Camponotus. Messor structor, belonging to the subfamily Myrmicinae, lives mainly in grasslands, in dry areas, and near human-inhabited villages. It has the characteristics of collecting plant seeds as food reserves, peeling seeds, and storing seeds in a nest. There are three sizes of worker ant: large, medium, and small. Large worker ants are usually responsible for carrying food, defending, and killing. In order to observe the load-carrying morphology of ants, the large worker ants in black-gold Turkish Camponotus and Messor structor were collected for research.

Black-gold Turkish Camponotus and Messor structor were collected from Xinjiang Province, China (73°40′–96°18′ E, 34°25′–48°10′ N). The ants were kept in tubes under long-day conditions (25 ± 1 °C; 50% RH) at the College of Biological and Agricultural Engineering Jilin University, Changchun, China.

### 2.2. Ants in Static Morphology

In the experiments, only adult worker ants are used. Specifically, 10 samples are randomly selected from the two types of ants collected (black-gold Turkish Camponotus and Messor structor). The samples are washed with water, sorted, and stored in 80% alcohol. Then, the samples are dried at room temperature for 12 h. Finally, all the samples are identified and counted.

Digital image acquisition and leg length measurements are performed using a Canon EOS 750 D digital camera (Canon, Tokyo, Japan) attached to a 3D electron microscope Jt-h800 system (Sunny, Changchun, Jilin, China).

For each ant, five standard linear measurements are taken using a visual micrometer mounted on the 3D electron microscope, and the average value is taken to the accuracy of 0.01 mm: fore tibia length, fore femur length, middle tibia length, middle femur length, hind tibia length, and hind femur length (Xin, R., Changchun, Jilin, China).

**Figure 1:**
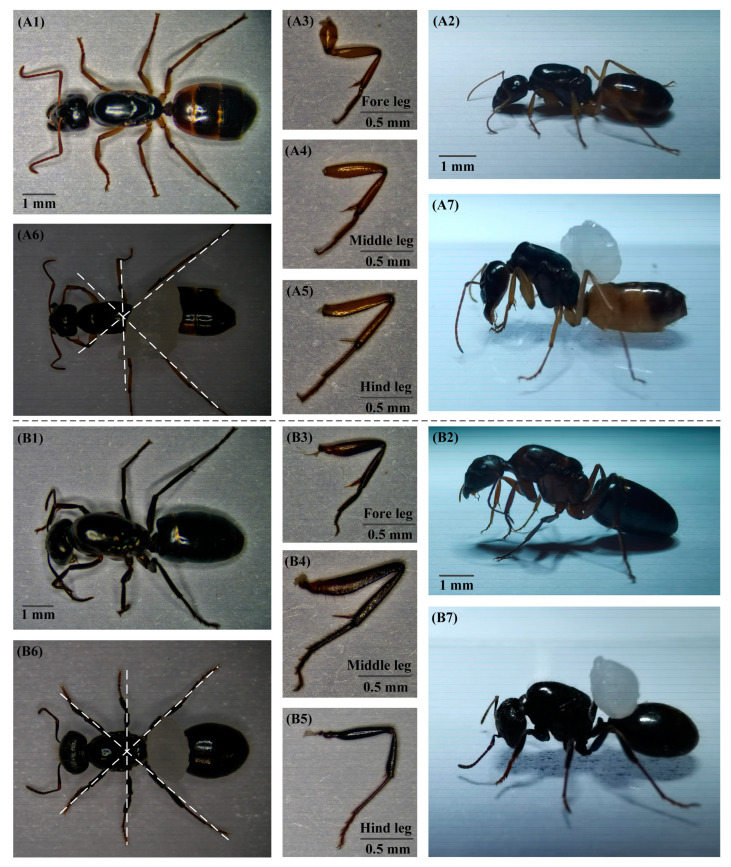
Body structure of ants. (**A1**) Top view of black-gold Turkish Camponotus in the static morphology. (**A2**) Side view of black-gold Turkish Camponotus in the static morphology. (**A3**) Magnified fore leg of black-gold Turkish Camponotus. (**A4**) Magnified middle leg of black-gold Turkish Camponotus. (**A5**) Magnified hind leg of black-gold Turkish Camponotus. (**A6**) Top view of black-gold Turkish Camponotus in the load-carrying morphology. (**A7**) Side view of black-gold Turkish Camponotus in the load-carrying morphology. (**B1**) Top view of Messor structor in the static morphology. (**B2**) Side view of Messor structor in the static morphology. (**B3**) Magnified fore leg of Messor structor. (**B4**) Magnified middle leg of Messor structor. (**B5**) Magnified hind leg of Messor structor. (**B6**) Top view of Messor structor in the load-carrying morphology. (**B7**) Side view of Messor structor in the load-carrying morphology.

According to the top and side views of the black-gold Turkish Camponotus (Figure 1(A1,A2)) and the Messor structor (Figure 1(B1,B2)) in the static morphology, we find that the lower limbs of ants are composed mainly of a femur and a tibia, with the same structure as the human femur and with a similar skeleton to that of human lower limbs. According to the measurement of sample data, the summary is as follows: The femur lengths of the fore legs of the black-gold Turkish Camponotus are 1.97–2.16 mm, while that of the Messor structor are 2.24–2.97 mm. The tibia lengths of the fore legs of the black-gold Turkish Camponotus are 3.17–3.88 mm, while that of the Messor structor are 3.59–4.77 mm. The femur lengths of the middle legs of the black-gold Turkish Camponotus are 1.98–2.36 mm, while that of the Messor structor are 2.97–3.26 mm. The tibia lengths of the middle legs of the black-gold Turkish Camponotus are 3.82–4.73 mm, while that of the Messor structor are 5.38–6.74 mm. The femur lengths of the hind legs of the black-gold Turkish Camponotus are 2.31–2.54 mm, while that of the Messor structor are 3.32–3.70 mm. The tibia lengths of the hind legs of the black-gold Turkish Camponotus are 4.62–6.25 mm, while that of the Messor structor are 7.52–8.69 mm. Moreover, we find that the fore legs of all the samples are the shortest (Figure 1(A3,B3)). The middle legs are relatively longer (Figure 1(A4,B4)). The hind legs of all the samples are found to be the longest (Figure 1(A5,B5)). Further, we find that the ratios of tibia to femur for all samples are 1.6, 1.8, 2.0, 2.2, and 2.5, respectively.

### 2.3. Ants in Load-Carrying Morphology

In this experiment, the load-carrying morphologies of the Black-gold Turkish Camponotus and the Messor structor are analyzed from video recordings. Video files are recorded using a Canon EOS 750 D digital camera (Canon, Tokyo, Japan) with a macro lens (Canon RF 100 mm F 2.8 L Macro IS USM, Canon, Tokyo, Japan; lens focal length: 100 mm) for about 1 min. In order to observe tiny details, the camera is set to 0.26 m extreme macro mode.

As exhibited in Figure 1(A6,B6), the left front leg and the right hind leg, the right front leg and the left hind leg, and the middle two legs are almost in straight lines for the two kinds of ants, so as to form a more stable state. For black-gold Turkish Camponotus, the angle between the femur and the tibia of the front and hind legs is approximately 45°, the angle between tibia of the front and hind legs and the horizontal axis is approximately 65°. The angle between the femur and tibia of the middle legs is approximately 30°, and the angle between tibia of the middle legs and the horizontal axis is approximately 70°.

Correspondingly, the angle between the femur and the tibia of the front and hind legs of the Messor structor is approximately 65°, and the angle between tibia of the front and hind legs and the horizontal axis is approximately 75°. Additionally, the angle between the femur and the tibia of the middle legs is approximately 45°, and the angle between tibia of the middle legs and the horizontal axis is approximately 80°.

## 3. Construction of Bio-Inspired Ant Lattice Structure

### 3.1. Design of Bio-Inspired Ant Lattice Structure

**Figure 2:**
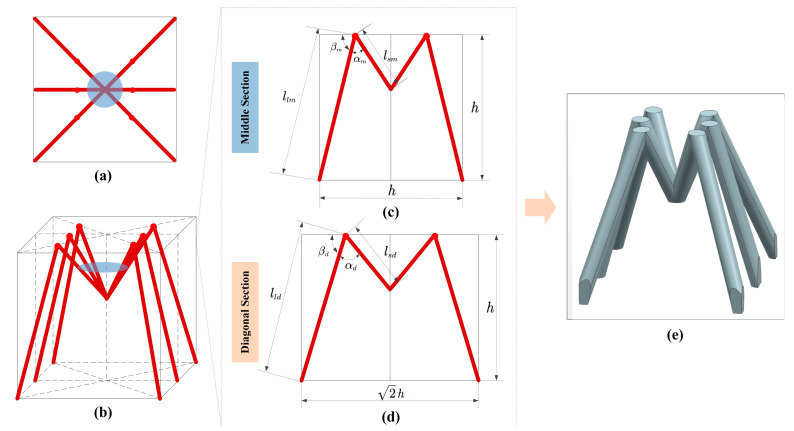
Bio-inspired ant lattice structure design. (**a**) Top view of 3D parameterized model, (**b**) three-dimensional drawing of parameterized model, (**c**) parameters of middle section, (**d**) parameters of diagonal section, and (**e**) three-dimensional drawing of bio-inspired ant lattice structure.

Inspired by ants, the lattice structure is designed with a total of 12 sides, including six short sides and six long sides, as depicted in Figure 2a,b. The short side *l_s_* corresponds to the femur of an ant, and the long side *l_l_* corresponds to the tibia of an ant. The left front leg and the right hind leg, on one hand, and the right front leg and the left hind leg, on the other, are respectively on the diagonal section of the cube, and the left middle leg and right middle leg are on the middle section of the cube. In order to simplify the model, it is assumed that the left front leg and the right hind leg, on one hand, and the right front leg and the left hind leg, on the other, have the same parameters: the length of the short side *l_sd_*, the length of the long side *l_ld_*, the angle between the short side and the long side *α_d_*, and the angle between the long leg and the horizontal axis *β_d_*, where the subscript *d* represents the diagonal section, as exhibited in Figure 2d. Correspondingly, the short side, the long side, the angle of the left middle leg and the right middle leg, and the angle between the long leg and the horizontal axis are *l_sm_*, *l_lm_*, *α_m_* and *β_m_*, respectively, where the subscript *m* represents the middle section, as exhibited in Figure 2c.

For the diagonal section, based on the Pythagorean theorem, the following equation is satisfied:

(1) $$l_{ld}\cos\beta_{d}+l_{sd}\cos(\pi -\alpha_{d}-\beta_{d})=\frac{\sqrt{2}}{2}h$$

For the middle section, based on the Pythagorean theorem, the following equation is satisfied:

(2) $$l_{lm}\cos\beta_{m}+l_{sm}\cos\left(\pi -\alpha_{m}-\beta_{m}\right)=h$$

where *h* represents the macroheight of the ant lattice.

According to the research on the static morphology of the Black-gold Turkish Camponotus and the Messor structor, the ratio of the long side to the short side *l_l_*/*l_s_* can be taken as 1.6, 1.8, 2.0, 2.2, and 2.5. According to the research on the load-carrying morphology pf the Black-gold Turkish Camponotus and the Messor structor, the diagonal section the angle between the short side and the long side *α_d_* can be taken as 45° and 65°, respectively, and the equivalent angle between the long leg and the horizontal axis *β_d_* can be taken as 65° and 75°, respectively. For the middle section, the angle between the short side and the long side *α_m_* can be taken as 30° and 45°, respectively, and the equivalent angle between the long leg and the horizontal axis *β_m_* can be taken as 70° and 80°, respectively. In addition, two random ratios of the long leg to the short leg, 2.7 and 3.0, respectively, are introduced for comparison. Based on the above data, 14 bionic lattice structures are established. According to the ratio of length change, the angle between the short side and the long side in the diagonal section, the equivalent angle between the long leg and the horizontal axis in the diagonal section, the angle between the short side and the long side in the middle section, and the equivalent angle between the long leg and the horizontal axis in the middle section, 14 lattice structures are defined: FBTC1, FMS1, FBTC2, FMS2, MBTC1, MMS1, MBTC2, MMS2, HBTC1, HMS1, RBTC1, RMS1, RBTC2, and RMS2. The specific parameters of the 14 lattice structures are shown in Table 1.

### 3.2. Finite Element Analysis

**Figure 3:**
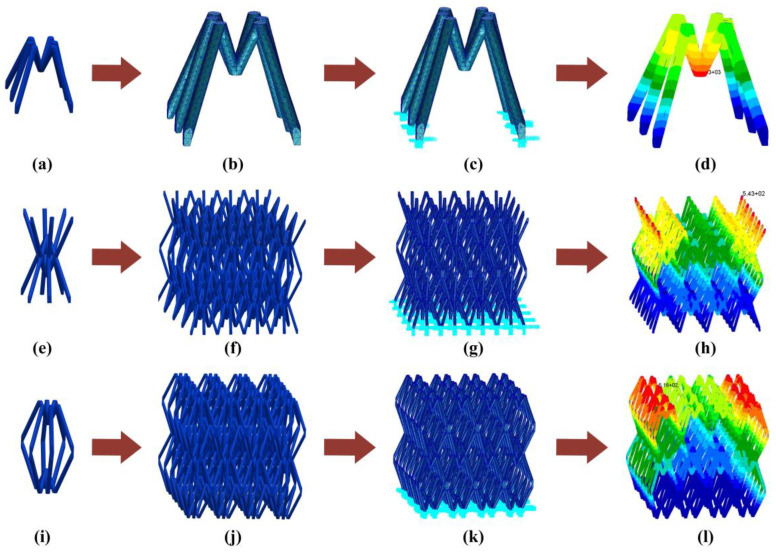
Finite element models of bio-inspired ant lattice structure. (**a**) Bio-inspired ant cellular structure, (**b**) finite element model of bio-inspired ant cellular structure, (**c**) constraints of finite element model of bio-inspired ant cellular structure, (**d**) first-order mode shape of bio-inspired ant cellular structure, (**e**) the top-to-top lattice structure, (**f**) finite element model of the top-to-top lattice structure, (**g**) constraints of finite element model of the top-to-top lattice structure, (**h**) first-order mode shape of the top-to-top lattice structure, (**i**) the foot-to-foot lattice structure, (**j**) finite element model of the foot-to-foot lattice structure, (**k**) constraints of finite element model of the foot-to-foot lattice structure, and (**l**) first-order mode shape of the foot-to-foot lattice structure.

In this chapter, the stability of these lattices is verified by conducting a simulation analysis. First, the stability of the unit is tested. The finite element modeling of these 14 units is carried out by using the commercial software MSC/Patran [44], as shown in Figure 3b. In order to ensure the accuracy of the calculation, the mesh size of 0.5 mm is selected for modeling. The boundary conditions are uniformly set to full constraints at the ends of the six long sides in Figure 3c. The same material is used for the finite element model of these units.

According to Table 1, under the same load-bearing form (when the values of *α* and *β* are constant), the fundamental frequency of these units increases with the increase in the ratio of long legs to short legs. When the ratio of long legs to short legs reaches 2.5, the fundamental frequency of the unit reaches the maximum. After that, with the increase in the ratio of long legs to short legs, the fundamental frequency of the unit gradually decreases. Correspondingly, when the ratio of long legs to short legs is constant, the fundamental frequency under the first load-bearing form is higher. By comparison, the fundamental frequency of the ninth HBTC1 unit is the highest.

Further studies are conducted on the HBTC1 unit. The unit can form two lattice structures by different stacking methods, including a top-to-top lattice structure (Figure 3e) and a foot-to-foot lattice structure (Figure 3i). Both of these lattice structures are modeled by using the commercial software MSC/Patran. In order to ensure the accuracy of the calculation, a mesh size of 0.5 mm is selected for modeling, as shown in Figure 3f,j. The boundary conditions of the top-to-top lattice structure are uniformly set to be fully constrained at the ends of the six long sides, as shown in Figure 3g. The boundary conditions of the foot-to-foot lattice structure are applied at the junction of the long and short legs for full constraints, as shown in Figure 3k. Similarly, the same material is chosen for the two lattice structures. A modal analysis is performed on the finite element model of the two lattice structures by using the commercial software MSC/Nastran. The first-order fundamental frequency of the top-to-top lattice structure is 2374 Hz, and the first-order fundamental frequency of the foot-to-foot lattice structure is 1607 Hz, as shown in Figure 3h,l. By comparison, the stability of the top-to-top lattice structure of the HBTC1 unit is better.

### 3.3. Manufacture of Bio-Inspired Lattice Structure

**Figure 4:**
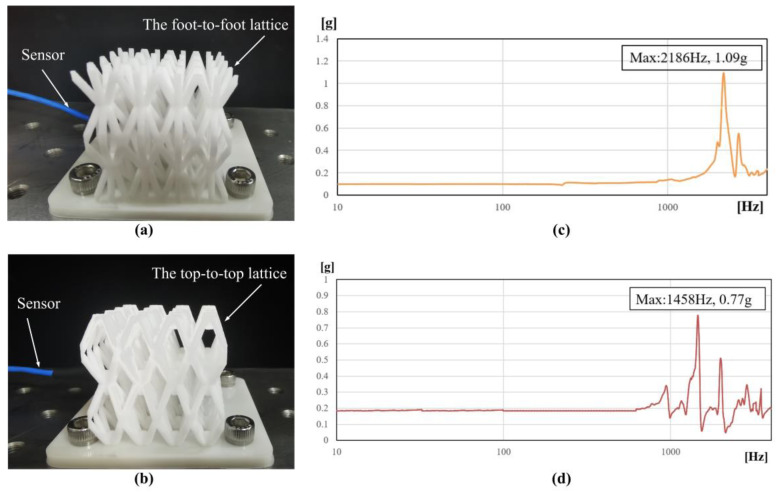
Vibration experiment. (**a**) The top-to-top lattice, (**b**) sweeping curve of the top-to-top lattice, (**c**) the foot-to-foot lattice, and (**d**) sweeping curve of the foot-to-foot lattice.

The top-to-top lattice and the foot-to-foot lattice (size: 40 mm × 40 mm × 40 mm) of the HBTC1 unit are additively manufactured by using the FS403P platform. In order to unify the mechanical properties of bio-inspired lattice structures and ensure the consistency principle of structure comparison, the same parameters are used when the bio-inspired lattice samples are manufactured by additive manufacturing, and the 3D printing parameters are recorded, as shown in Table 2. Two samples of the top-to-top lattice and the foot-to-foot lattice are exhibited in Figure 4.

### 3.4. Experimental Verification

The acceleration test is used to verify the structural stiffness and the accuracy of simulation results between the top-to-top lattice and the foot-to-foot lattice of the HBTC1 unit. The result of the fundamental frequency is obtained according to the acceleration test. The two lattices are manufactured by additive manufacturing. The test piece is fixed on the platform by using four screws on the white adapter, and the micro three-way sensor is arranged on the side of the test piece with glue to collect the acceleration data, as exhibited in Figure 4. The first-order fundamental frequencies of the two lattice structures are determined according to the peak points of the sweep frequency curves, which are 2186 Hz and 1458 Hz, respectively. According to Table 3, the error rates of the simulation results and the experimental results are 8.0% and 9.2%, respectively. Contrasted with the simulation result, the reason for the low experimental results may be due to the process problems of additive manufacturing. The results of both the simulation analysis and the stiffness experiment show that the stiffness of the top-to-top ant lattice of the HBTC1 unit is better.

The compression experiment is carried out on the universal tensile-testing machine (KQL computer-controlled electronic universal testing machine, Liu, R., Changchun, Jilin, China) at room temperature (RT). The data on force and displacement during the loading process are transmitted to the computer from sensors mounted on the testing machine. Their experimental compression velocity is 1 mm/min.

**Figure 5:**
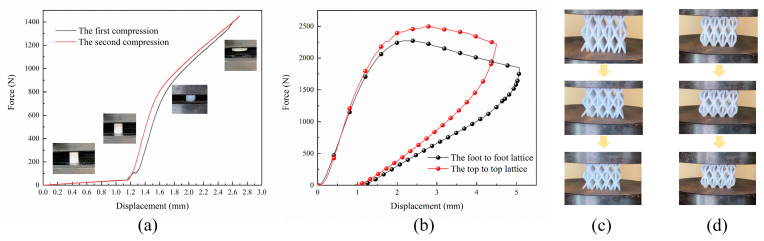
Compression experiment. (**a**) Material compression experiment diagram; (**b**) compression experiment diagram of two samples; (**c**) compression process of the top-to-top lattice sample at the displacement of 0.5 mm, 1 mm, and 1.5 mm; and (**d**) compression process of the foot-to-foot lattice sample at the displacement of 0.5 mm, 1 mm, and 1.5 mm.

Figure 5a depicts the force-displacement curve of nylon material compression for vibration simulations. It can be seen from the compression results in Figure 5b that the peak load of the top-to-top lattice is 245.8 N higher than that of the foot-to-foot lattice, indicating that the top-to-top lattice has extremely high structural stiffness and strength. In addition, it can be seen in Figure 5c that in the initial deformation stage of the structure, the foot-to-foot lattice shows a supporting effect in the outward deformation and has a strong anti-deformation ability, while in Figure 5d, the top-to-top lattice shows a lateral bending in the deformation and is prone to deformation from the deformation mode. Therefore, the top-to-top lattice of the HBTC1 unit is selected to fill the solid mixture structure, and the feasibility of this method is verified.

## 4. Application of Bio-Inspired Lattice Structure in the Support Structure of the Fengyun-3 Satellite Payload

### 4.1. The Original Design of the Support Structure

**Figure 6:**
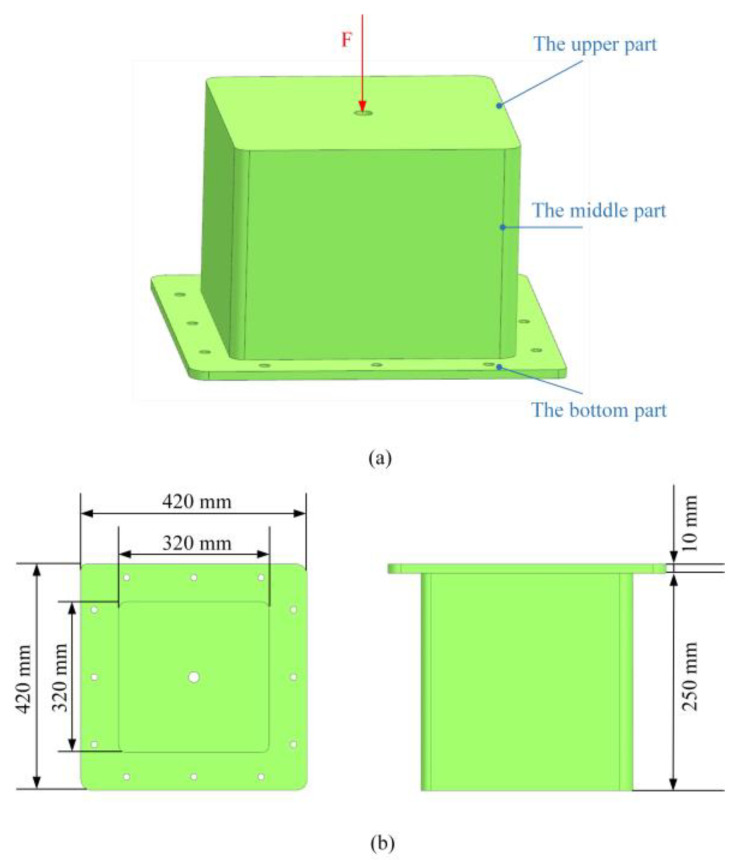
The support structure with load and boundary conditions and geometric dimensioning. (**a**) Load and boundary condition and (**b**) geometric dimensioning.

The support structure is designed for connecting the horizontal support and the vertical support; this connection plays an important role in the design of the Fengyun-3 satellite payload. The upper part of the structure is fixed with vertical support by initiating explosive devices, and the bottom part is connected with horizontal support by using screws. The geometric features and dimensions are shown in Figure 6. The thickness of the upper and bottom parts is 10 mm, and the thickness of the middle part is 10 mm.

**Figure 7:**
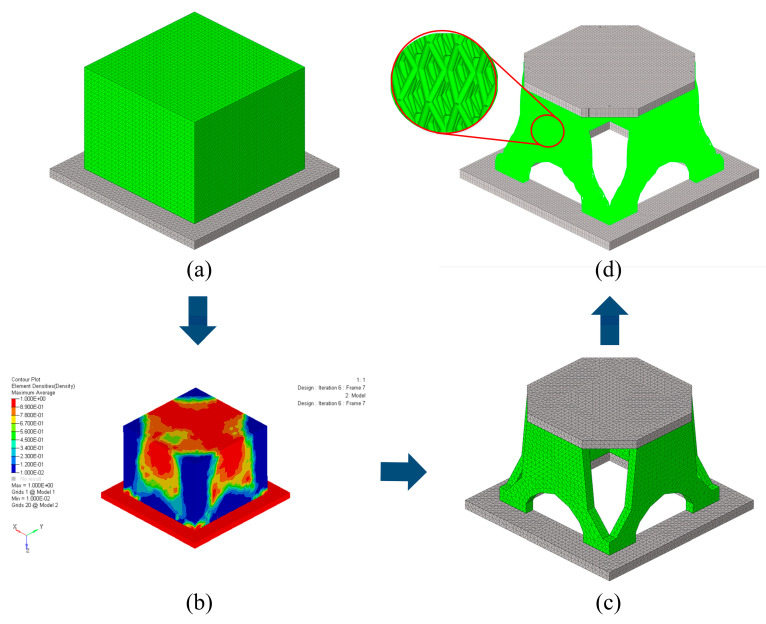
Design and optimization process of the solid-lattice hybrid support structure. (**a**) The finite element model of the initial design, (**b**) the topology optimization result, (**c**) the reconstructed model of the topology optimization model, and (**d**) the solid-lattice hybrid optimization model.

The finite element model of the support frame is established, which can be broken down into 49,315 elements and 13,202 nodes, as shown in Figure 7a. In order to save computational costs and ensure a high-quality mesh model, these tiny bolt holes are ignored in the process of building the finite element model. Because the bottom part of the support structure is connected to the horizontal support, it is assumed that the bottom part is fixed. The mesh nodes near the position of the screw holes are fully constrained as the boundary condition, and the concentrated force, F = 20 kN, is applied at the center of the upper part along the vertical direction. It is assumed that a common aluminum alloy-7A09 is used to manufacture the support structure. The basic properties of 7A09 are shown in Table 4. The total mass of the support structure is 5.02 kg. The first-order fundamental frequency of the structure is 305.3 Hz, obtained through modal analysis.

### 4.2. The Optimal Design of the Support Structure

In order to achieve the goal of lightweight design, a four-step procedure is carried out to design and optimize the solid-lattice hybrid structure inspired by ants, by combining topology optimization, bio-inspired lattice structure filling, and size optimization. First of all, the initial optimization model is obtained through topology optimization to pursue the optimal material distribution. The model is divided into two parts: the gray area is the nondesignable area, and the green area is the designable area. They are illustrated in Figure 7a. A 30% volume fraction constraint is set for optimization, and the optimization objective is to maximize the fundamental frequency. The topology optimization result with a symmetry constraint is depicted in Figure 7b. Second, the model is manually divided according to the distribution of pseudodensity values (*ρ_i_*). Minimum pseudodensity regions (*ρ_i_* < 0.1) are removed, low pseudodensity regions (0.1 ≤ *ρ_i_* ≤ < 0.7) are converted into lattice filled parts, and high pseudodensity regions (*ρ_i_* ≤ 0.7) are kept as solid parts. In addition to the distribution of the pseudodensity value, the influence of the main bearing path should also be considered. At the same time, in order to convert this optimization result into an actual engineering structure, some minor details need to be ignored. The reconstructed model, the total mass of which is 4.21 kg, is exhibited in Figure 7c. Subsequently, according to the reconstructed model, a solid-lattice hybrid model is established, in which four Y-shaped supporting parts are filled with ant lattices. Finally, size optimization has been carried out to obtain the optimal cross-sectional area of the lattice structure under specified loads and constraints. To ensure that the final design meets the weight requirements, a mass constraint of 4.2 kg is introduced in the size-optimization problem. The final optimization model of the solid-lattice hybrid model is illustrated in Figure 7d. The mass of the solid-lattice hybrid model is 3.43 kg.

### 4.3. Mechanical Performance Verification of the Solid-Lattice Hybrid Structure

The solid-lattice hybrid FE model is also completed by using the commercial software MSC/Patran. In order to ensure the accuracy of the calculation, a mesh size of 1 mm is selected for modeling. The boundary conditions are uniformly set to be fully constrained at the position of the screw holes, the same as the original design.

**Figure 8:**
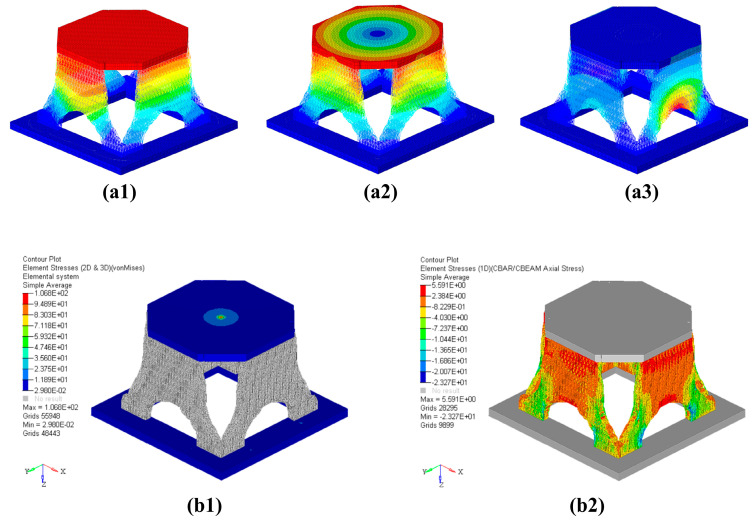
Simulation analysis result graph. (**a1**) First-order mode diagram, (**a2**) second-order mode diagram, (**a3**) third-order mode diagram, (**b1**) von Mises stress distribution of 3D elements of the reconstructed solid-lattice hybrid structure, and (**b2**) von Mises stress distribution of 1D elements of the reconstructed solid-lattice hybrid structure.

The modal analysis of the reconstructed solid-lattice hybrid model is carried out by using MSC/Nastran. As shown in Figure 8(a1–a3), the first three fundamental frequencies of the solid-lattice hybrid structure are 290.6 Hz, 291.4 Hz, and 438.3 Hz. Compared with the initial design model, the mass of the optimized model decreases by 32%, but the first-order fundamental frequency decreases by only 4.8%. It is proved that this solid-lattice hybrid structure inspired by ants has excellent stiffness characteristics for a lightweight structure.

Furthermore, it is necessary to verify the strength of the structure. In order to ensure the safety of aerospace structures, under the yield limit load conditions, the structural parts of the products should have a positive safety margin *M.S.*, and *M.S.* is defined as follows [45]:

(3) $$M.S.=\frac{\left[\sigma \right]}{\sigma_{MAX}\cdot f}-1$$

where *σ* represents the yield limit, *σ_MAX_* represents the maximum stress generated by the identification load, and *f* represents the safety factor, which is generally taken as 1.2.

For the reconstructed solid-lattice hybrid model, a stress analysis is performed under the prescribed load and boundary conditions to verify the strength of the structure. The von Mises stress distribution of the reconstructed solid-lattice hybrid model is shown in Figure 8(b1,b2). The maximum value is 106.8 MPa, occurring at the place where the concentrated force is applied. The stress distribution of the rest of the structure is uniform, and the value is small. For the reconstructed solid-lattice hybrid model, the safety margin *M.S.* = 2.67, indicating that the reconstructed solid-lattice hybrid structure has good strength characteristics on the basis of ensuring a lightweight structure. It also proves the feasibility of this multiscale optimization method, inspired by ants, to ensure that aerospace structures are lightweight.

## 5. Conclusions

In order to further improve the effect of a lightweight structure, this paper proposed a bionic design method of providing lattice structure filling for the support structure of the Fengyun-3 satellite payload inspired by ants. In order to realize this bionic design, we proposed a parameterized design method providing of lattice structure based on an ant-leg configuration. In order to compare the stiffness and strength performance, a modal analysis and a stress analysis were carried out on the original design model and the reconstructed optimization model. The conclusion is summarized as follows:

(1) In view of the research on the leg configuration characteristics of two classic ants, namely the Black-gold Turkish Camponotus and the Messor structor, regarding their static morphology and load-carrying morphology, it can be seen that the HBTC1 cell has the highest stiffness among the 14 cells, according to the simulation analysis.

(2) The two stacking methods of the HBTC1 cell, namely the top-to-top lattice structure and the foot-to-foot lattice structure, were simulated and tested. It was found that the top-to-top lattice structure had better stiffness and anticompressibility, which was selected to fill the solid mixture structure.

(3) The multiscale optimization method, which combined topology optimization, bio-inspired lattice structure filling, and size optimization, was applied to the lightweight design of the support structure of the Fengyun-3 satellite payload. Compared with the initial design model, the mass of the solid-lattice hybrid optimized model decreased by 32%, but the first-order fundamental frequency decreased by only 4.8%. Meanwhile, the safety margin of the solid-lattice hybrid optimized model was 2.67, which was greater than zero. This indicated that the reconstructed solid-lattice hybrid structure had good stiffness and strength characteristics to ensure a lightweight structure.

## Figures and Tables

**Table 1:** The specific parameters of bionic cell structures.

No.	Units	*l_l_*/*l_s_*	*α_d_*	*β_d_*	*α_m_*	*β_m_*	Fundamental Frequency
1	FBTC1	1.6	45°	65°	30°	70°	3564
2	FMS1	1.6	65°	75°	45°	80°	3145
3	FBTC2	1.8	45°	65°	30°	70°	4713
4	FMS2	1.8	65°	75°	45°	80°	4342
5	MBTC1	2.0	45°	65°	30°	70°	5326
6	MMS1	2.0	65°	75°	45°	80°	4832
7	MBTC2	2.2	45°	65°	30°	70°	5946
8	MMS2	2.2	65°	75°	45°	80°	5453
9	HBTC1	2.5	45°	65°	30°	70°	6794
10	HMS1	2.5	65°	75°	45°	80°	6287
11	RBTC1	2.7	45°	65°	30°	70°	6024
12	RMS1	2.7	65°	75°	45°	80°	5461
13	RBTC2	3.0	45°	65°	30°	70°	5277
14	RMS2	3.0	65°	75°	45°	80°	4795

The outer envelope size of all units is 10 × 10 × 10 mm^3^, and the macroheight of the ant lattice *h* = 10 mm. Note: (1) fore black-gold Turkish Camponotus 1 cell structure, (2) fore Messor structor 1 cell structure, (3) fore black-gold Turkish Camponotus 2 cell structure (4) fore Messor structor 2 cell structure, (5) middle black-gold Turkish Camponotus 1 cell structure, (6) middle Messor structor 1 cell structure, (7) middle black-gold Turkish Camponotus 2 cell structure, (8) middle Messor structor 2 cell structure, (9) hind black-gold Turkish Camponotus 1 cell structure, (10) hind Messor structor 1 cell structure, (11) random black-gold Turkish Camponotus 1 cell structure, (12) random Messor structor 1 cell structure, (13) random black-gold Turkish Camponotus 2 cell structure, and (14) random Messor structor 2 cell structure.

**Table 2:** FS403P 3D-printing parameters.

Parameters	Scanning Speed (mm/s)	Build Cavity Temperature (°C)	Laser Power (W)	Preheating Temperature (°C)	Jumping Speed (mm/s)	Material
Tensile sample	7.62	169	22	140	2.54	Nylon

**Table 3:** Comparison of small-scale characteristic sweep test results and simulation results.

	The Top-to-Top Lattice	The Foot-to-Foot Lattice
Low-level vibration test results/Hz	2186	1458
Simulation results/Hz	2374	1607
Error rate	8.0%	9.2%

**Table 4:** Properties of selected aluminum alloy.

Material	Aluminum Alloy
Brand	7A09
Young’s modulus (E)	71 GPa
Poisson’s ratio (m)	0.33
Density (ρ)	2700 kg/m^3^
Yield limit	470 MPa

## Data Availability

Not applicable.
